# Progress in Cardiac Conduction Disease and the Emergence of Artificial Intelligence in Epidemiological Research

**DOI:** 10.1016/j.jacadv.2024.101007

**Published:** 2024-06-05

**Authors:** Harold L. Kennedy

**Affiliations:** Department of Medicine and Cardiovascular Disease, University of Missouri School of Medicine, Columbia, Missouri, USA

**Keywords:** cardiac conduction disease, digital data integration, epidemiology

**Figure:**
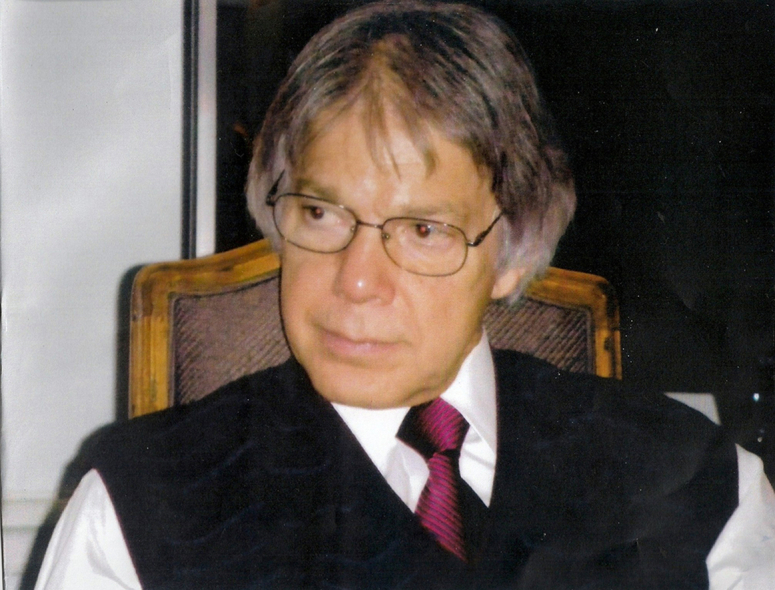

Like many achievements in medicine, science, or medical innovations, one must pause and reflect upon the contribution of Haimovich et al^1^ for their rigorous advancement of epidemiologic research into the electronic health records of a large multi-institutional primary care health care system during the time period of 2001 to 2019. The inclusion/exclusion criteria and median 10-year follow-up methodology represent an admirable contribution to the study of cardiac conduction disease, which has progressed over both decades and centuries.^2^ The current study calls attention to the now active integration and ongoing evolution within the current decade of various academic disciplines of cardiovascular medicine and results in combining efforts in cardio-metabolic disease, cardio-oncologic disease, cardio-genetic, and now cardio-epidemiologic research. Moreover, it focuses attention on changes that occurred in medical research during the 19-year longitudinal period of the study. The digital world emerged in such a blinding fashion; it dampens somewhat the advances of our past studies.

Currently, the pace of science and medical research are clearly embedded in the digital age, and the reader must weigh the contribution of a longitudinal epidemiological study over approximately 2 decades within newly appreciated medical perspectives and variables. Medical knowledge does not change every 10, 5, or even 2 years as in the past, but currently embraces emerging digital technologies at an ever-quickening pace within a year. We must examine the study findings within this context.

The authors analyzed the Community Cohort Project of a primary care cohort of 208,045 patients with an existing baseline 12-lead electrocardiogram (ECG) and, after rigorously excluding those with cardiac devices or changes due to prior cardiothoracic surgery, followed them longitudinally from 2001 to 2019.^1^ The authors sought to categorize the prevalence and incidence of cardiac conduction disease with more precision than previous reports and identify clinical risk factors for conduction system disease across a broad range of known cardiac conduction abnormalities. Their data, clear illustrations, and outcomes are interesting and well presented. However, within the “fading candle of time,” and with all such progress come some unintended findings and results.^3^ Risk factors obtained at the start of follow-up included hypertension, diabetes, chronic kidney disease, coronary artery disease, myocardial infarction, atrial fibrillation, heart failure, valvular disease, and stroke/transient ischemic attack. The authors note that their records did not contain body mass index or obesity data for adjustment, during which time it is now known to affect 40% of the U.S. population and several comorbidities. This undoubtedly affected their population.^4^^,^^5^ Moreover, the geographic location of the Community Cohort Project in a New England geographic health care system known for academic excellence brings our consciousness to thoughts of our current epidemiologic focus on evaluating equity imposed on study populations by an appreciation of the variability of the social determinants of disease (eg, air quality, economic level, access to health care, and so on) that exist throughout the United States. As the authors noted, conduction disorders “may be even more common in populations with greater comorbidity burden and less ready access to care.”^1^ Nevertheless, their data described the prevalence of conduction disorders as 27% among men and 15% among women. Over a median period of 10 years, the incidence of conduction disorders occurred more frequently in men (8.78 events/1,000 person-years) vs women (4.34/1,000 person-years), and multivariable analysis found older age (HR: 1.25 per 5 years: [95% CI: 1.24-1.26]) and myocardial infarction (HR: 1.39 [95% CI: 1.26-1.54] to be the most important clinical risk factors associated with incident conduction disease.^1^ Their findings of observing a 20-fold increased higher prevalence of conduction disease and an increased incidence rate from earlier reports are consistent with their improved methodology of the study, the aging of the population, and the social determinants of the population studied. Notwithstanding the limitations of the electronic health records in existence and confounding risk factor variables of such a large population, the take-home message of a greater prevalence of conduction disease in men as compared to women and the variables of age and myocardial infarction identified those most at risk of cardiac conduction incident events in this New England cohort of primary care patients.

In the study of medicine throughout the ages, we recognize that we all “stand on the shoulders” of those who came before us.^3^ This has been very true over the centuries in the study of cardiac conduction disease. First came the anatomical discovery of the cardiac conduction system starting with Jan Evangelista Purkinje describing Purkinje fibers in 1839.^2^ There then followed a cascade of further anatomical studies by Walter Gaskell, Willhelm His Jr, Sunoro Tawara, Arthur Keith, Martin Flack, and Kasel Wenckebach extending to the end of the decade and early 1900s, which defined the structures of the cardiac conduction system.^2^ Then emerged the early recordings of electrical activity by Agustus Waller, the string galvanometer ECG by William Einthoven, and the followers of Sir Thomas Lewis, William Craib, and Frank Wilson defining the electrocardiogram to display abnormalities of cardiac conduction.^6^^,^^7^ These advances were extended further in the last century during the emergence of the long-term electrocardiogram by Jeffrey Holter and a plethora of innovative physicians, engineers, and scientists who contributed to our understanding of conduction disorders and arrhythmias.^3^

Currently, in 2024, we stand on the brink of a digital advance that can only be described in its pace as a quantum light-speed movement of medical knowledge facilitated by the realm and promise of artificial intelligence (AI).^8^ Even early in the current century, the esteemed physician Hein JJ Wellens called attention to identifying small changes in the conduction of the QRS to determine heart failure,^9^ and the importance of the genetic influence on cardiac conduction blocks were recognized.^10^ The organization of AI deep-learning models is underway to try and place standardized scientific reporting guidelines,^11^ but nevertheless, there already exist emerging AI ECG reports to predict mechanisms of sudden cardiac arrest, heart failure, and atrial fibrillation (Figure 1).^12^, ^13^, ^14^

**Figure 1:**
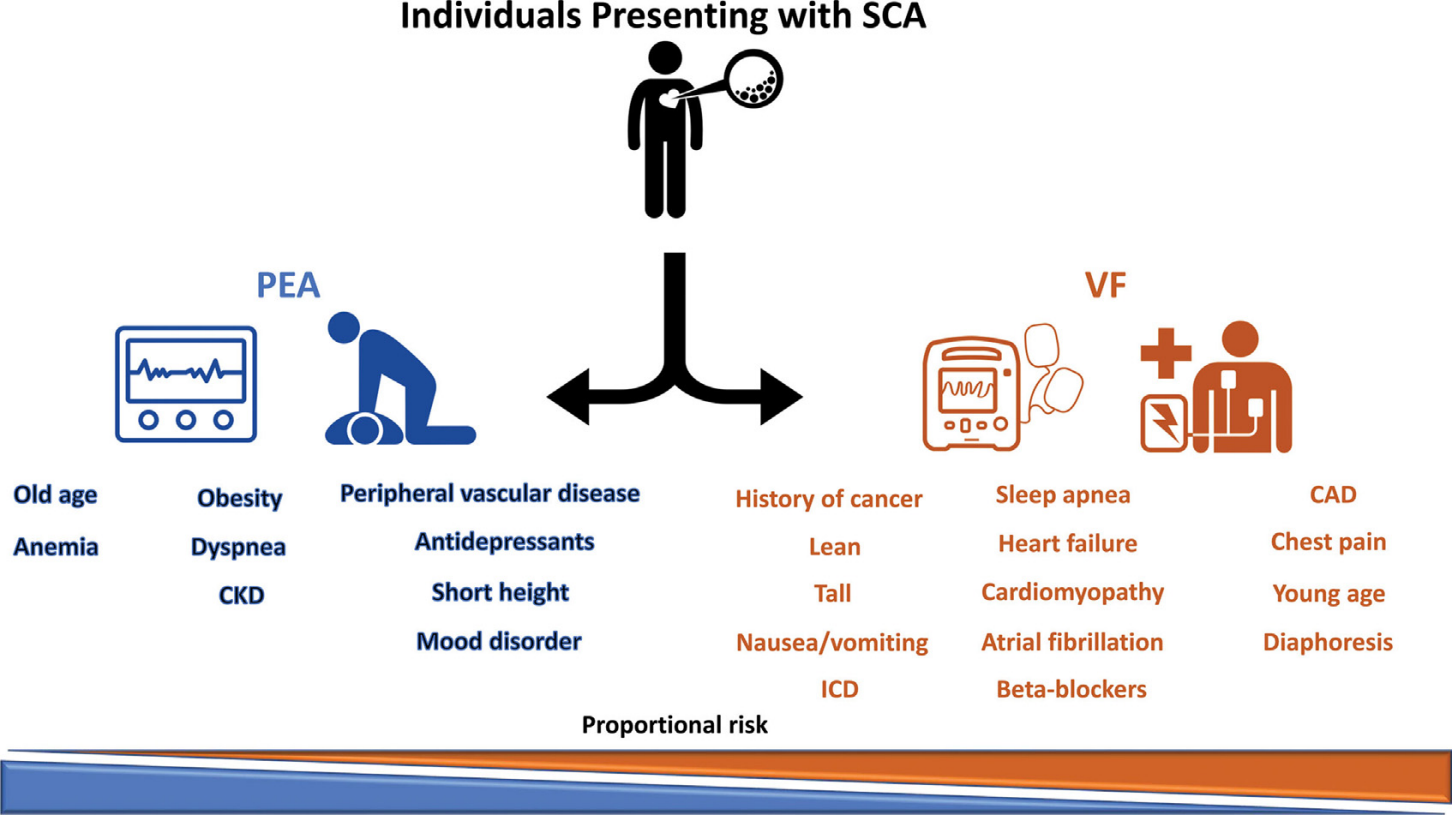
**Schematic Illustration of the Key Determinants of PEA-SCA and VF-SCA** CAD = coronary artery disease; CKD = chronic kidney disease; ICD = implantable cardioverter defibrillator; PEA = pulseless electric activity; SCA = sudden cardiac arrest; VF = ventricular fibrillation. Included with permission: artificial intelligence model predicts sudden cardiac arrest manifesting with pulseless electric activity vs ventricular fibrillation. Reprinted with permission from Holmstrom et al.^12^

Epidemiologic advance in the digital world of the United States and the United Kingdom took a marked development in the last 2 to 3 years with the emergence of the modern digital techniques employed at the U.S. Census Bureau and the UK Biobank. The U.S. Census data, which addresses every county and state in the United States with modern digital collective methods, will have a great influence on future studies accounting for the effects of the social determinants of disease to measure health equity of the U.S. population and direct government policy.^15^ Adjustments for economics of the population, access to health care, air pollution quality, geographic location, and a myriad of both known and yet unknown or unrecognized risk factors will be possible from such data. Thus, cardiovascular-epidemiologic studies of populations will be adjusted with more precise data, rendering better clinical prediction and leading to better preventive methods. This is an exciting developing saga that the cardiovascular community must now embrace and realize that we are all involved in both cardiovascular clinical care as well as the epidemiology of public health and the well-being of populations.

Epidemiological longitudinal studies will continue to be needed going forward with regards to establishing outcome data, but their preciseness and accuracy will be greatly modified by a host of variables contained in the large language models of AI, currently known or unknown, or heretofore unrecognized factors. AI adds to the robustness of all data, and we must ensure its proper investigation.

## Funding support and author disclosures

This paper has been funded by the Cardiovascular Research Foundation. The author has reported that they have no relationships relevant to the contents of this paper to disclose.
